# BST-2 Promotes N Protein Degradation and Inhibits Viral Replication Through the MARCHF8/NDP52 Autophagy Pathway

**DOI:** 10.3390/microorganisms13081865

**Published:** 2025-08-09

**Authors:** Chenchen Zhao, Yan Qin, Haixin Huang, Yuying Li, Xinyu Zhang, Lin Zhou, Lulu Xie, Yimin Zhou, Yanqing Hu, Wei Chen, Tian Lan, Wen-Chao Sun

**Affiliations:** Wenzhou Key Laboratory for Virology and Immunology, Institute of Virology, Wenzhou University, Wenzhou 325035, China; zhaochenchen0508@163.com (C.Z.); qinyan_qy@163.com (Y.Q.); huanghaixinn@163.com (H.H.); 15858559633@163.com (Y.L.); j648260369@gmail.com (X.Z.); q103937513@outlook.com (L.Z.); lxl_13750811496@163.com (L.X.); 24451041042@stu.wzu.edu.cn (Y.Z.); huyanqing0811@163.com (Y.H.); 23451039005@stu.wzu.edu.cn (W.C.)

**Keywords:** BST2/tetherin, SADS-CoV, MARCHF8, NDP52

## Abstract

Swine acute diarrhea syndrome coronavirus (SADS-CoV) is a recently discovered enteric coronavirus that has caused considerable economic losses in the pig industry. SADS-CoV was first reported in 2017 in Guangdong Province, China, and subsequently in Fujian, Guangxi, Henan and Jiangxi Provinces. Bone marrow stromal cell antigen 2 (BST-2), also known as tetherin, acts as an antiviral protein to limit the release of a wide range of enveloped viruses. However, the relationship between BST-2 and SADS-CoV has rarely been studied. Here, we showed that endogenous BST-2 expression is downregulated by SADS-CoV infection in Vero-E6 and ST cells by 2- to 3-fold. The overexpression of BST-2 inhibited SADS-CoV replication, whereas the knockdown of the BST-2 gene in Vero cells restored SADS-CoV replication. Further study revealed that BST-2 targets the SADS-CoV nucleocapsid protein (N) and decreases N protein expression, and that the BST-2 transmembrane (TM) domain is essential for this activity. Moreover, the degradation of the SADS-CoV N protein promoted by BST-2 is mediated by the membrane-associated ring-CH-type finger 8 (MARCHF8)/calcium binding and coiled-coil domain 2 (NDP52) autophagosome pathway. Overall, we found that BST-2 suppresses viral proliferation by inducing the breakdown of the SADS-CoV N protein via the MARCHF8/NDP52 pathway.

## 1. Introduction

Swine acute diarrhea syndrome coronavirus (SADS-CoV) is part of the *Alphacoronavirus* genus and the *Orthocoronavirinae* subfamily of the *Coronaviridae* family [1]. The SADS-CoV genome is approximately 27 kilobases (kb) long and closely genetically related to the *Rhinolophus* (bat) coronavirus strain HKU2, with nucleotide homology exceeding 90% [2,3]. SADS-CoV causes diarrhea and severe dehydration in piglets less than 1 week of age, with a mortality rate exceeding 90%. Furthermore, research has indicated that SADS-CoV has zoonotic potential [4]. Among coronaviruses, the nucleocapsid (N) protein is the most highly conserved structural protein. Previous studies have reported that N proteins are involved in various immune response-related processes of the host, such as apoptosis and autophagy, and antagonize the host’s innate immune responses [5]. The SADS-CoV N protein can inhibit the IFN-β promoter activity induced by TBK1 and its upstream key signaling molecules (TRAF3, IPS-1, RIG-I, and MDA5) [6]. The SADS-CoV N protein can also block the phosphorylation and nuclear translocation of IRF3 and NF-κB induced by polyinosine-cytidine (PolyI:C), interact with RIG-I independent of its RNA binding activity, and induce K27-, K48-, and K63-linked ubiquitination. It leads to proteasome-dependent degradation and can inhibit the production of IFN-β [7].

Bone marrow stromal cell antigen 2 (BST-2) (also known as tetherin, CD137, HM1.24) is a type II transmembrane protein induced by type I or type II IFNs [8]. Its topological structure includes an N-terminal domain, a transmembrane domain composed of an α-helix, a coiled extracellular domain (ED), and a C-terminal glycosylphosphatidylinositol (GPI) anchoring region [9]. BST-2 can inhibit the release of most enveloped viruses, including human immunodeficiency virus 2 (HIV-2) [10], ebolavirus (EBOV) [11], hepatitis B virus (HBV) [12], chikungunya virus (CHIKV) [13], and severe acute respiratory syndrome coronavirus 2 (SARS-CoV-2) [14]. BST-2 interacts with virions, which then either remain on the cell surface or undergo endocytosis and ubiquitin-dependent degradation [15]. BST-2 has also been shown to alter the effects of IFN-I, NF-κB, and T-cell effector functions in immune regulation. Type I IFN reduces the versatility of Vpu by upregulating BST-2, thereby reducing its ability to protect HIV-1-infected cells from natural killer (NK) cell responses [16]. BST-2 recruits the E3 ubiquitin ligase MARCHF8 to catalyze the formation of lysine (K27)-linked polyubiquitin chains on mitochondrial antiviral signaling proteins (MAVSs) and relies on NDP52 to transport it to the autophagosome, degrading the MAVS and thereby negatively regulating type I IFNs [17]. BST2 mediates porcine epidemic diarrhea virus (PEDV) restriction by recruiting MARCHF8 to catalyze the ubiquitination of the PEDV-encoded nucleocapsid protein [18].

In the present study, we show that BST-2 constrains viral replication in cells challenged with SADS-CoV and further demonstrate that BST-2 triggers the degradation of the SADS-CoV N protein via the MARCHF8/NDP52 autophagic pathway. Collectively, these data provide a novel understanding of the process by which BST-2 restricts viral replication via the selective autophagy-dependent breakdown of the N protein.

## 2. Methods Summary

### 2.1. Cell Culture and Viruses

Human embryonic kidney (HEK-293T, ATCC, CRL-3216, Manassas, VA, USA), swine testis (ST, ECACC, 92040221, Porton Down, UK), and African green monkey kidney (Vero-E6, ATCC, CRL-1586) cells were maintained in DMEM (HyClone, C11995500BT, Logan, UT, USA) supplemented with 10% FBS (CELLMAX, SA211.02, Stockholm, Sweden). SADS-CoV was propagated in Vero-E6 cells in our laboratory and diluted in trypsin-supplemented DMEM (10 mg/mL). During the incubation process, the cell plate was shaken at intervals of approximately 30 min, and then the incubation was continued. Following 12 h of transfection or 6 h of viral inoculation, the drug was added to the cells at a specific concentration, after which samples were collected as scheduled in the experimental plan.

### 2.2. Reagents and Primers

The sources of the major reagents used are listed in Table 1. The primers were synthesized by Sangon Biotech (Table 2). The identity of each recombinant plasmid was confirmed by Sanger sequencing to ensure accuracy.

### 2.3. Western Blot Analysis

The cells were collected at specific time points. The cells were washed twice with precooled PBS, and lysis buffer (P0013, Beyotime Biotechnology, Haimen, China) was added. The lysates were mixed with 6× SDS-PAGE loading buffer (P0015, Beyotime, China) and denatured by heating for 10 min. For Co-IP, the cells were lysed in buffer containing a protease inhibitor. Protein isolation was performed via TransGen Biotech’s ProteinIso^®^ Protein A/G Resin (DP501, Beijing, China). The target antibody was added to the supernatant, and the resulting mixture was heated to 100 °C for 5 min to induce denaturation before Western blotting.

Proteins were separated by SDS-PAGE on Vazyme gels (E304-01, Nanjing, China) and electroblotted onto PVDF membranes. Following blocking, the samples were incubated with specific primary antibodies, and chemiluminescent bands were detected via an enhanced chemiluminescence kit (P10300, NCMbiotech, Suzhou, China). Protein band intensities were quantified with ImageJ software (version 1.8).

### 2.4. RNA Extraction and qPCR

RNA was extracted from cell or virus samples using a Sangon Biotech kit (B511321, Shanghai, China), and subsequent cDNA synthesis was achieved using TaKaRa reverse transcriptase (RR036A, Kusatsu, Japan). For RT-qPCR, the reactions were performed in triplicate using a Vazyme kit (Q712, China) and run on an ABI QuantStudio 3 platform.

### 2.5. Confocal Immunofluorescence

HEK-293T cells were fixed with 4% paraformaldehyde (P1110; Solarbio, Beijing, China) for 20 min, after which they were permeabilized for 1 h to facilitate antibody access. The indicated antibodies were applied to the samples, and the nuclei were labeled with DAPI (C1006; Beyotime, China). Images were acquired using an inverted fluorescence microscope configured for multichannel imaging.

### 2.6. Statistical Analysis

The data shown are representative of three independent experiments. Statistical evaluations were performed via one-way ANOVA and two-tailed Student’s *t* test using GraphPad Prism(version 8.0.2) (GraphPad Software, San Diego, CA, USA). In all analyses, *p* values of <0.05 were considered to indicate statistical significance.

## 3. Results

### 3.1. BST-2 Inhibits SADS-CoV Replication

**Figure 1:**
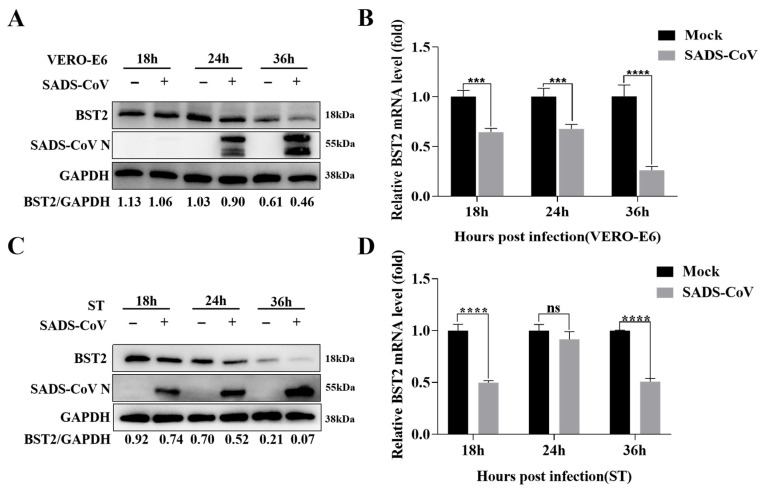
Endogenous BST-2 expression is inhibited by SADS-CoV. (**A**,**B**) Vero-E6 cells were subjected to SADS-CoV infection (MOI = 1) or mock infection and then collected at specified time points for Western blotting and RT-qPCR. (**C**,**D**) ST cells were subjected to SADS-CoV infection (MOI = 1) or mock infection and then collected at specified time points for Western blotting and RT-qPCR. The data are presented as the means ± SDs of triplicate experiments. ***, *p* < 0.001, ****, *p* < 0.0001 (two-tailed Student’s *t* test).

Although BST-2 is widely recognized for its antiviral activities, its role in SADS-CoV pathogenesis remains underexplored. To characterize the interaction of BST-2 with SADS-CoV, we analyzed BST-2 expression in ST and Vero-E6 cells. As shown in Figure 1, endogenous BST-2 expression at both the protein and mRNA levels was lower in SADS-CoV-infected cells than in uninfected cells.

**Figure 2:**
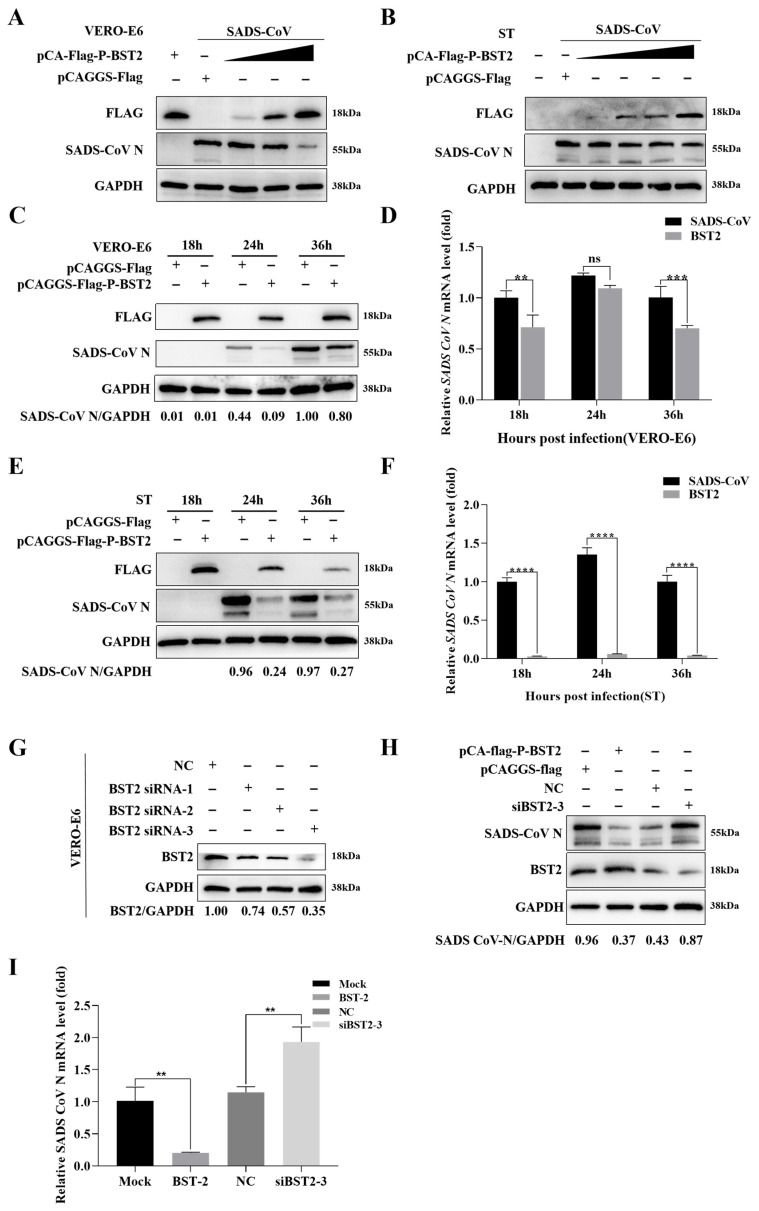
BST-2 restricts SADS-CoV replication. (**A**,**B**) The cells were transfected for 12 h with different concentrations of the BST-2 plasmid. The cells were subsequently infected with SADS-CoV (MOI of 1 or 10). GAPDH served as the internal control for sample loading. (**C**,**D**) Vero-E6 cells were treated with either BST-2 plasmid or an empty vector for 12 h and subsequently infected with SADS-CoV (MOI = 1). The cells were then collected at the specified time points. Western blotting and RT-qPCR were used to analyze the lysates. (**E**,**F**) ST cells were treated with either the BST-2 plasmid or an empty vector for 12 h and subsequently infected with SADS-CoV at an MOI of 10. Western blotting and RT-qPCR were used to analyze the lysates. (**G**) Vero-E6 cells were treated with either a negative control (NC) or one of three different types of BST-2 siRNAs for 24 h. Western blotting was used to analyze the cell lysates, and GAPDH served as the internal control for sample loading. (**H**,**I**) Vero-E6 cells were transfected with the mock plasmid, BST-2 plasmid, NC siRNA or siRNA-2 for 24 h and then infected with SADS-CoV (MOI = 1). Western blotting and RT-qPCR were used to analyze the lysates. The data are presented as the means ± SDs of triplicate experiments. “ns”, no statistical significance; “**”, *p* < 0.01; “***”, *p* < 0.001; “****”, *p* < 0.0001 (two-tailed Student’s *t* test).

A Flag-tagged BST-2 plasmid was subsequently generated to assess the impact of BST-2 overexpression on SADS-CoV replication. SADS-CoV replication was inhibited by BST-2 in a concentration-dependent manner (Figure 2A,B). Next, we examined the effect of BST-2 on viral replication at distinct time intervals following infection. As anticipated, overexpression of BST-2 led to decreased expression of viral proteins and mRNAs in infected cells across multiple time points following infection (Figure 2C–F). These findings indicate that BST-2 restricts the replication of SADS-CoV.

We engineered siRNAs targeting BST-2 to confirm these conclusions (Figure 2G). As shown in Figure 2H,I, silencing BST-2 in cells led to significant restoration of high viral protein and mRNA levels. Overall, these findings indicate that BST-2 restricts SADS-CoV replication.

### 3.2. The BST-2 TM Domain Is Essential for the Inhibition of the SADS-CoV N Protein

**Figure 3:**
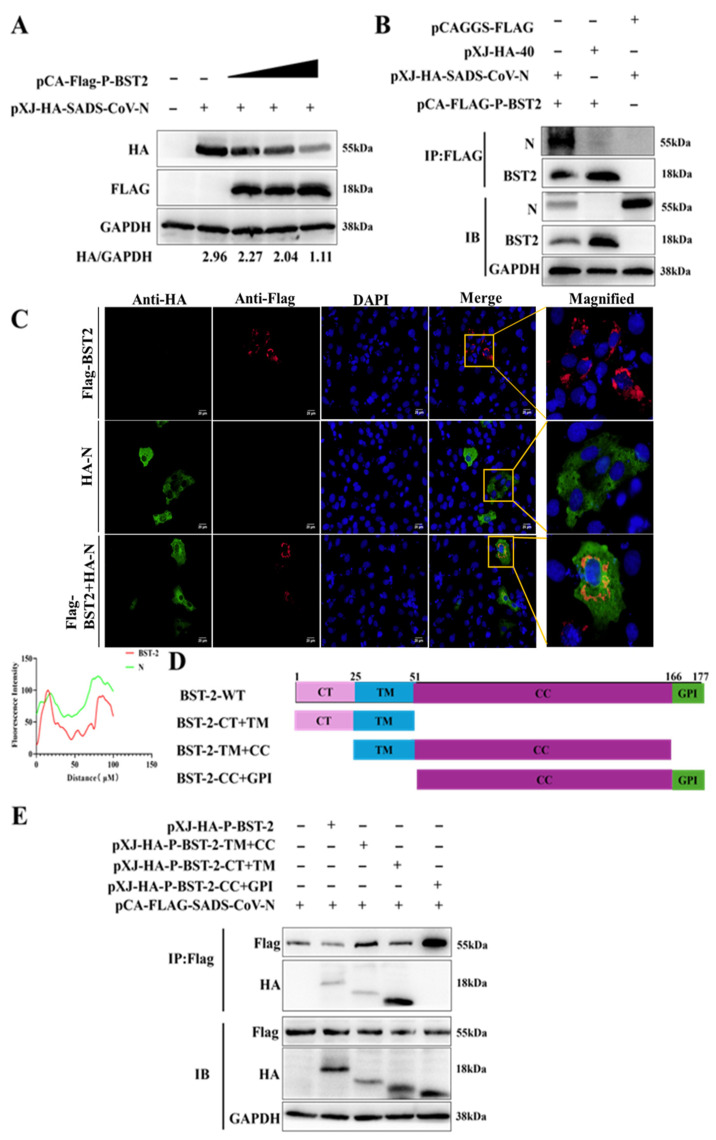
The domains of BST-2 that interact with the SADS-CoV N protein. (**A**) HEK293T cells were transfected with the BST-2 and N plasmids for 24 h, and the lysates were analyzed by Western blotting. (**B**) HEK-293T cells were transfected with BST-2, N or empty vectors for 24 h. The lysates were then analyzed via co-IP. (**C**) Vero-E6 cells were transfected with BST-2, N or empty vector for 24 h. The cells were fixed and labeled with DAPI. Fluorescence was observed with a confocal immunofluorescence microscope. Scale bars: 20 µm. (**D**) Domain map of BST-2. (**E**) HEK293T cells were transfected with plasmids encoding BST-2 full-length or truncated BST-2 in combination with the N protein plasmid for 24 h. The lysates were then analyzed via co-IP.

Although BST-2 is recognized to inhibit SADS-CoV replication, the underlying mechanism remains incompletely understood. Our analysis (Figure 3A) revealed that the N protein level decreased in a dose-dependent manner in response to increasing BST-2 expression. Similarly, co-IP experiments revealed that BST-2 coimmunoprecipitated with the SADS-CoV N protein (Figure 3B). Additionally, confocal microscopy and immunofluorescence staining experiments confirmed that BST-2 and the SADS-CoV N protein were colocalized in the cytoplasm (Figure 3C). Although we cannot exclude the possibility that the overexpression of BST-2 may affect its localization, these data directly demonstrate that BST-2 targets the SADS-CoV N protein and promotes its degradation.

We further investigated the region of BST-2 responsible for N protein inhibition by constructing three BST-2 deletion mutants: CT+TM, TM+CC, and CC+GPI (Figure 3D). HEK293T cells were transfected with HA-BST-2-expressing plasmids, HA-BST-2 mutant-expressing plasmids, or N plasmids. Subsequent analysis indicated that full-length BST-2, BST-2-CT+TM, and BST-2-TM+CC bound to the N protein to varying degrees (Figure 3E). In addition, the level of BST-2-CT+TM expression was markedly greater than that of the other treatments. These data indicate that the BST-2 TM domain is the most important region for the inhibition of the SADS-CoV N protein.

### 3.3. BST-2 Facilitates Degradation of the SADS-CoV N Protein Through Activation of the BST-2/MARCH8/NDP52-Mediated Autophagosome Pathway

**Figure 4:**
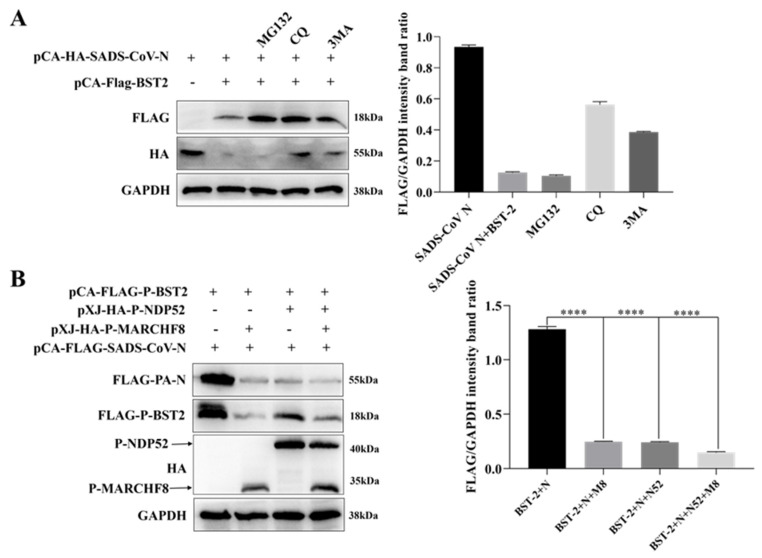
BST-2 promotes SADS-CoV N protein degradation through the autophagosome pathway. (**A**) HEK293T cells were transfected with plasmids encoding BST-2 and N for 18 h and then treated with MG132 (5 µM), CQ (10 µM), or 3MA (0.5 µM) for 12 h. (**B**) HEK-293T cells were cotransfected with N and BST-2, MARCHF8 or NDP52 for 24 h. The cell lysates were collected for analysis via Western blotting. The data are presented as the means ± SDs of triplicate experiments. “****”, *p* < 0.0001 (two-tailed Student’s *t* test).

Considering the above observations, we next aimed to determine the molecular mechanism through which BST-2 inhibits the replication of SADS-CoV. We selected the proteasome inhibitor MG132, the autophagosome formation inhibitor 3-methyladenine (3MA), and the autolysosome inhibitor CQ for follow-up experiments. The data revealed that the two autophagy inhibitors 3MA and CQ blocked the degradation of the N protein facilitated by BST-2 (Figure 4A), suggesting that the autophagy–lysosome pathway may be involved in mediating N protein degradation.

**Figure 5:**
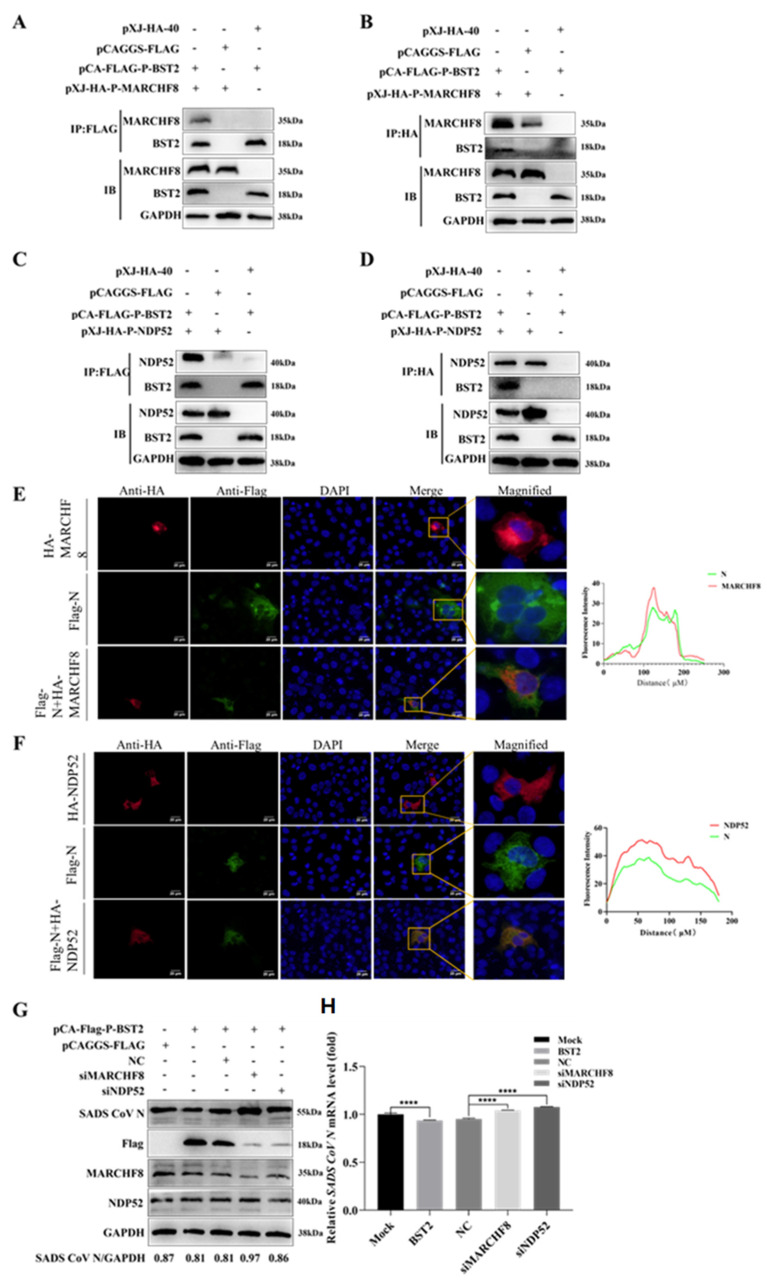
BST-2 promotes SADS-CoV N protein degradation through the MARCHF8/NDP52 pathway. (**A**,**B**) HEK-293T cells were transfected with plasmids encoding BST-2, MARCHF8 or an empty vector. The lysates were then analyzed via co-IP. (**C**,**D**) HEK-293T cells were transfected with BST-2, NDP52, or empty vector for 24 h. The lysates were then analyzed via co-IP. (**E**,**F**) Vero-E6 cells were transfected with NDP52, MARCHF8, N, or empty vector for 24 h. The cells were fixed and processed for immunofluorescence staining. Fluorescence was observed under a confocal immunofluorescence microscope. Scale bars: 20 µm. (**G**,**H**) Following cotransfection with BST-2 and either the empty vector, NC siRNA, MARCHF8 siRNA, or NDP52 siRNA for 12 h, the cells were infected with SADS-CoV at an MOI of 1. The data are presented as the means ± SDs of triplicate experiments. ****, *p* < 0.0001 (two-tailed Student’s *t* test).

Research has confirmed that BST-2 induces viral protein degradation by recruiting E3 ligases, leading us to hypothesize that BST-2 promotes N protein degradation via the selective autophagy pathway. Therefore, building on the above results, we explored the mechanism by which BST-2 promotes N protein degradation with a focus on the MARCHF8/NDP52 pathway. To assess the involvement of MARCHF8 and NDP52, we transfected HEK-293T cells with plasmids expressing BST-2, MARCHF8, NDP52, or N proteins. As shown in Figure 4B, the expression of exogenous MARCHF8 and NDP52 promoted the degradation of the N protein. Moreover, co-IP experiments revealed that BST-2 interacted with MARCHF8 and NDP52 (Figure 5A–D). Through confocal microscopy and immunofluorescence staining experiments, we observed that MARCHF8 or NDP52 colocalized with the SADS-CoV N protein in the cytoplasmic region (Figure 5E,F). Next, we engineered siRNAs targeting MARCHF8 or NDP52. Following transfection with plasmids encoding these siRNAs or BST-2 for 24 h, Vero-E6 cells were infected with SADS-CoV. The findings revealed that siMARCHF8- and siNDP52-transfected cells expressed more SADS-CoV N protein than did NC siRNA-transfected cells (Figure 5G,H). Furthermore, in future experiments, reintroducing MARCHF8 or NDP52 after knockout may help us quickly determine the role of MARCHF8 or NDP52 in viral infection and may promote the inhibition of SADS-CoV replication by BST2 [17]. In summary, these observations demonstrate that BST-2 facilitates the degradation of the SADS-CoV N protein through the activation of the MARCH8/NDP52-dependent autophagy pathway.

## 4. Discussion

Selective autophagy is a process in which damaged or unnecessary organelles within cells are degraded through the lysosomal system. Several previous reports indicate that selective autophagy plays an important role in antiviral activity [19]. In brief, the virus infects the host, and viral particles or viral proteins are labeled with ubiquitin or galactose lectin tags. Selective autophagy receptors (SARs), such as P62, NDP52, or OPTN, subsequently bind to ubiquitinated viral particles or viral proteins through their ubiquitin-binding domain UBD motif. The LIR domain of SARs binds to the LC3 protein attached to the autophagosome membrane and ultimately fuses with lysosomes to degrade the bound pathogen [20].

Host cells can use autophagy to inhibit viral replication, but some viruses can also use autophagy to promote viral replication. For example, the sequestosome 1 (SQSTM1/p62) receptor can bind to the dengue virus (DENV) capsid protein to induce autophagy and inhibit virus replication [21]. FUBP3 inhibits the replication of PEDV by degrading the N protein through the MARCHF8/NDP52 selective autophagy pathway [22]. The ICP0 and Ca^2+^-mediated proteasomal receptor downregulates the expression of p62 and OPTN and promotes the escape of the autophagy defense mechanism at the early stage of HSV-I infection [23], and classical swine fever virus (CSFV) inhibits the expression of nuclear dot protein 52 kDa (NDP52/CALCOCO2) by mediating the ubiquitination and SUMO of NDP52 through the PTEN-induced kinase 1 (PINK1)-Parkin pathway [24].

Studies have shown that SADS-CoV induces autophagy through the IRE1–JNK–Beclin1 pathway [25] and the ITGA3/Akt/mTOR signaling pathway [26] to promote replication. However, the antiviral mechanisms of autophagy remain unclear. Our study demonstrated that both PABPC4 [27] and BST-2 act as antiviral proteins to restrict viral replication through the selective autophagy pathway. As an RNA-binding protein, PABPC4 relies primarily on its RNA recognition motif (RRM) domains to engage in mRNA metabolism, whereas BST-2, a transmembrane protein, depends on its TM domain to tether virions. The antiviral roles of PABPC4 and BST-2 share a conserved function in targeting the N protein via MARCHF8/NDP52, whereas their distinct modes of action—RNA binding for PABPC4 versus transmembrane tethering for BST-2—highlight divergent strategies in antiviral immunity.

In this study, we confirmed the molecular mechanism by which BST-2 inhibits SADS-CoV at the cellular level. However, this study has certain limitations, including the lack of in vivo experiments and unresolved issues regarding cell line dependence. In addition, some issues that need to be further addressed, such as the specific ubiquitination sites on the N protein recognized by MARCHF8 and the precise role of N protein ubiquitination, require further research. As indicated by current research, only PEDV and SADS-CoV share the same mechanism, but the existence of this mechanism in other coronaviruses remains unconfirmed. The broad-spectrum applicability of this mechanism among coronaviruses warrants further investigation. If such broad-spectrum applicability is confirmed, it could become a vital target in the prevention, control, and treatment of zoonotic diseases.

In summary, our study revealed that BST-2 forms a complex with the SADS-CoV N protein and promotes its degradation through selective autophagy, thereby inhibiting the replication of SADS-COV. These findings may help elucidate the mechanism by which selective autophagy inhibits SADS-CoV and provide a new target for the study of SADS-CoV treatment.

## 5. Conclusions

To date, the mechanisms by which SADS-CoV evades host immune surveillance are poorly understood. Our study revealed that BST-2 expression is downregulated in SADS-CoV-infected cells. Mechanistic investigations further revealed that BST-2 inhibits viral replication through autophagy-mediated degradation of the SADS-CoV nucleocapsid (N) protein. These findings lay a foundation for deciphering the host-virus interaction networks involved in antiviral defense and viral immune evasion.

## Figures and Tables

**Table 1:** Summary of reagents.

Antibody or Chemical	Manufacturer	Country
anti-BST-2	Proteintech	Wuhan, China
anti-GAPDH	Proteintech	Wuhan, China
anti-MARCHF8	Proteintech	Wuhan, China
anti-NDP52	Proteintech	Wuhan, China
anti-HA	Bioss	Beijing, China
anti-Flag	Bioss	Beijing, China
Chloroquine	Aladdin	Shanghai, China
MG132	Aladdin	Shanghai, China
3-Methyladenine	Aladdin	Shanghai, China

**Table 2:** Primers.

Name	Sequence (5′–3′)
pCAGGS-Flag-P-BST2 sense	gatgacgacgataaggaattcATGTCACCTAGTTTGT ATTCCTACAGC
pCAGGS-Flag-P-BST2 antisense	attaagatctgctagctcgagTCAGGTCAGCAGGGCA TTGA
si-BST2-1 sense	CCAUGGAUGACACUUGCAA
si-BST2-1 antisense	UUGCAAGUGUCAUCCAUGG
si-BST2-2 sense	GGGAGAUCACUACAUUGAA
si-BST2-2 antisense	UUCAAUGUAGUGAUCUCCC
si-BST2-3 sense	GGAGCGACUGAGAAGAGAA
si-BST2-3 antisense	UUCUCUUCUCAGUCGCUCC

## Data Availability

The original contributions presented in this study are included in the article. Further inquiries can be directed to the corresponding authors.
